# Lewis Acid Catalyzed Amide Bond Formation in Covalent Graphene–MOF Hybrids

**DOI:** 10.1021/acs.jpcc.3c01821

**Published:** 2023-06-29

**Authors:** Rabindranath Lo, Martin Pykal, Andreas Schneemann, Radek Zbořil, Roland A. Fischer, Kolleboyina Jayaramulu, Michal Otyepka

**Affiliations:** † Institute of Organic Chemistry and Biochemistry, Czech Academy of Sciences, v.v.i., Flemingovo nám. 2, 160 00 Prague 6, Czech Republic; ‡ Regional Centre of Advanced Technologies and Materials, Czech Advanced Technology and Research Institute, Palacký University Olomouc, Křížkovského 511/8, Olomouc 77900, Czech Republic; § Chair of Inorganic and Metal−Organic Chemistry, Department of Chemistry and Catalysis Research Centre, Technical University of Munich, 85748 Garching, Germany; ∥ Hybrid Porous Materials Lab, Department of Chemistry, Indian Institute of Technology Jammu, Jammu & Kashmir 181221, India; ⊥ IT4Innovations, VŠB-Technical University of Ostrava, 17. listopadu 2172/15, 70800 Ostrava-Poruba, Czech Republic; # Lehrstuhl für Anorganische Chemie I, Technische Universität Dresden, Bergstr. 66, 01069 Dresden, Germany; % Nanotechnology Centre, CEET, VSB, Technical University of Ostrava, 17. listopadu 2172/15, 70800 Ostrava-Poruba, Czech Republic

## Abstract

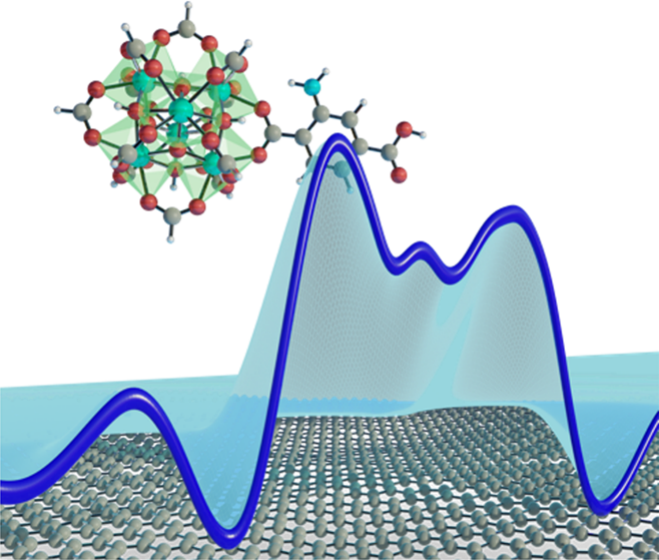

Covalent hybrids of graphene and metal–organic frameworks (MOFs) hold immense potential in various technologies, particularly catalysis and energy applications, due to the advantageous combination of conductivity and porosity. The formation of an amide bond between carboxylate-functionalized graphene acid (GA) and amine-functionalized UiO-66-NH_2_ MOF (Zr_6_O_4_(OH)_4_(NH_2_-bdc)_6_, with NH_2_-bdc^2–^ = 2-amino-1,4-benzenedicarboxylate and UiO = Universitetet i Oslo) is a highly efficient strategy for creating such covalent hybrids. Previous experimental studies have demonstrated exceptional properties of these conductive networks, including significant surface area and functionalized hierarchical pores, showing promise as a chemiresistive CO_2_ sensor and electrode materials for asymmetric supercapacitors. However, the molecular-level origin of the covalent linkages between pristine MOF and GA layers remains unclear. In this study, density functional theory (DFT) calculations were conducted to elucidate the mechanism of amide bond formation between GA and UiO-66-NH_2_. The theoretical calculations emphasize the crucial role of zirconium within UiO-66, which acts as a catalyst in the reaction cycle. Both commonly observed hexa-coordinated and less common hepta-coordinated zirconium complexes are considered as intermediates. By gaining detailed insights into the binding interactions between graphene derivatives and MOFs, strategies for tailored syntheses of such nanocomposite materials can be developed.

## Introduction

Metal–organic frameworks (MOFs) are a unique class of materials comprised of metal centers linked by multitopic organic ligands, forming three-dimensional (3D) or two-dimensional (2D) porous networks.^1^ Striking characteristics include high surface area, tunable pore structures, large pore volume, high redox activity, and tunable physicochemical properties, which make MOFs promising candidate materials for sorption and electrochemical applications.^2^ However, MOFs are challenged by issues associated with their limited chemical stability, poor electrical conductivity, and sometimes inaccessible, intricate pores.^3^ Hybridization of MOFs with graphene materials can be beneficial if the host structure provides appropriate interactions for stabilizing and improving the desired properties.^4^ Indeed, many recent efforts have focused on integrating functionalized graphene with MOFs by covalent and noncovalent approaches to result in hybrid materials with improved electrochemical and physicochemical properties, widening the scope toward various energy and environmental applications.^5^

In recent years, quantum mechanical electronic structure calculations on MOFs have revealed efficaciously their properties and functionalities.^6^ Computationally expensive electronic structure calculations have been utilized for the calculation of various physical and chemical properties, e.g., structural properties,^7^ bulk mechanical properties,^8^ magnetism,^9^ catalytic activity,^10^ binding energies,^11^ and gas adsorption sites,^12^ whereas comparatively less expensive classical force field methods combined with simulation have been usually used for the estimation of adsorption isotherms, isosteric heat of adsorption, and gas diffusion constants.^13−17^ Recently, computational studies on graphene–MOF hybrid materials to understand the interfacial growth of MOF nanoparticles on functionalized graphene surfaces have taken off. A series of zirconium-based MOFs having different geometric dimensions and surface charge properties were studied theoretically for the adsorption on graphene oxide (GO) surfaces in aqueous solution.^18^ The DFT calculations showed that the electrostatic attractions combined with π–π interactions, hydrogen bonding, and Lewis acid–base interactions were the main cause for the heteroaggregation between GO and Zr-based MOFs.

Our group has worked for the past decade on various graphene–MOF hybrid materials prepared through covalent and noncovalent experimental routes for CO_2_ storage, oil–water separation, water splitting, and energy storage/conversion applications.^5^ Recently we reported the covalent assembly of graphene acid (GA) with the amine-functionalized metal–organic framework Zr_6_(OH)_4_(O)_4_(NH_2_-bdc)_6_ (denoted as UiO-66-NH_2_, with NH_2_-bdc^2–^ = 2-amino-1,4-benzenedicarboxylate), via the amide bond.^19,20^ In contrast to graphene oxide, in GA most of the oxygen sites are located on the basal plane, allowing for better control of the chemical bonding of suitably functionalized MOFs between the graphene layers.^21^ Additionally, the strong bonding between each of the pristine components established a hierarchical pore architecture as well as significant conductivity, which are beneficial for rapid ion transportation to interaction sites (i.e., pendant functional groups) which drove the development of asymmetric supercapacitors and gas sensors.

Herein we have studied the mechanism of covalent assembly of GA with the amine-functionalized MOF using density functional theory (DFT) calculations. The direct formation of amides by condensing nonactivated carboxylic acids and amines is considered the most challenging due to the acid–base reaction which occurs between the acid and amine.^22,23^ At elevated temperature (80–160 °C), the ammonium carboxylate salt formation can be overcome, and amides can be formed in good yields. However, the high temperatures usually needed are not appropriate for highly functionalized or sensitive substrates and restrict the applicability of thermal amidation. Thus, using a catalyst is a smart approach to enable atom-economical formation of amides under mild reaction conditions. Generally, the amides are formed with the use of stoichiometric coupling reagents to activate as well as protect the carboxylic acid. For direct amidation, the most well-documented catalysts are boron or group IV metal complexes under mild conditions.^24−38^ The zirconium-catalyzed system, particularly ZrCl_4_ and ZrCp_2_Cl_2_, is cost-efficient, resulting in high conversions of the substrate using low catalyst loadings.^37,38^ However, the mechanistic pathway of amide formation with an amine-functionalized MOF and GA is yet obscure. A detailed outlining of the reaction mechanism would expand the fundamental understanding of the mechanism for this type of graphene–MOF hybrid material and, in turn, both allow the development of more efficient reaction protocols and offer evidence for future development of new catalysts. The all-atom classical molecular dynamics (MD) simulations have been used to describe the structure and stability of the hybrid of GA@UiO-66-NH_2_. Then, we present a detailed profiling of the reaction mechanism using the PBE0-D3/def2-TZVPP level of theory. The role of the zirconium catalyst in the amide formation has also been investigated through the possible alternative mechanism of direct amide reaction. In addition, the mechanistic investigations were also performed in the presence of a coordination modulator, 4-aminobenzoic acid. The aim of this research is to understand the covalent linkage and the influence of the amino groups during the catalytic reaction.

## Results and Discussion

**Figure 1:**
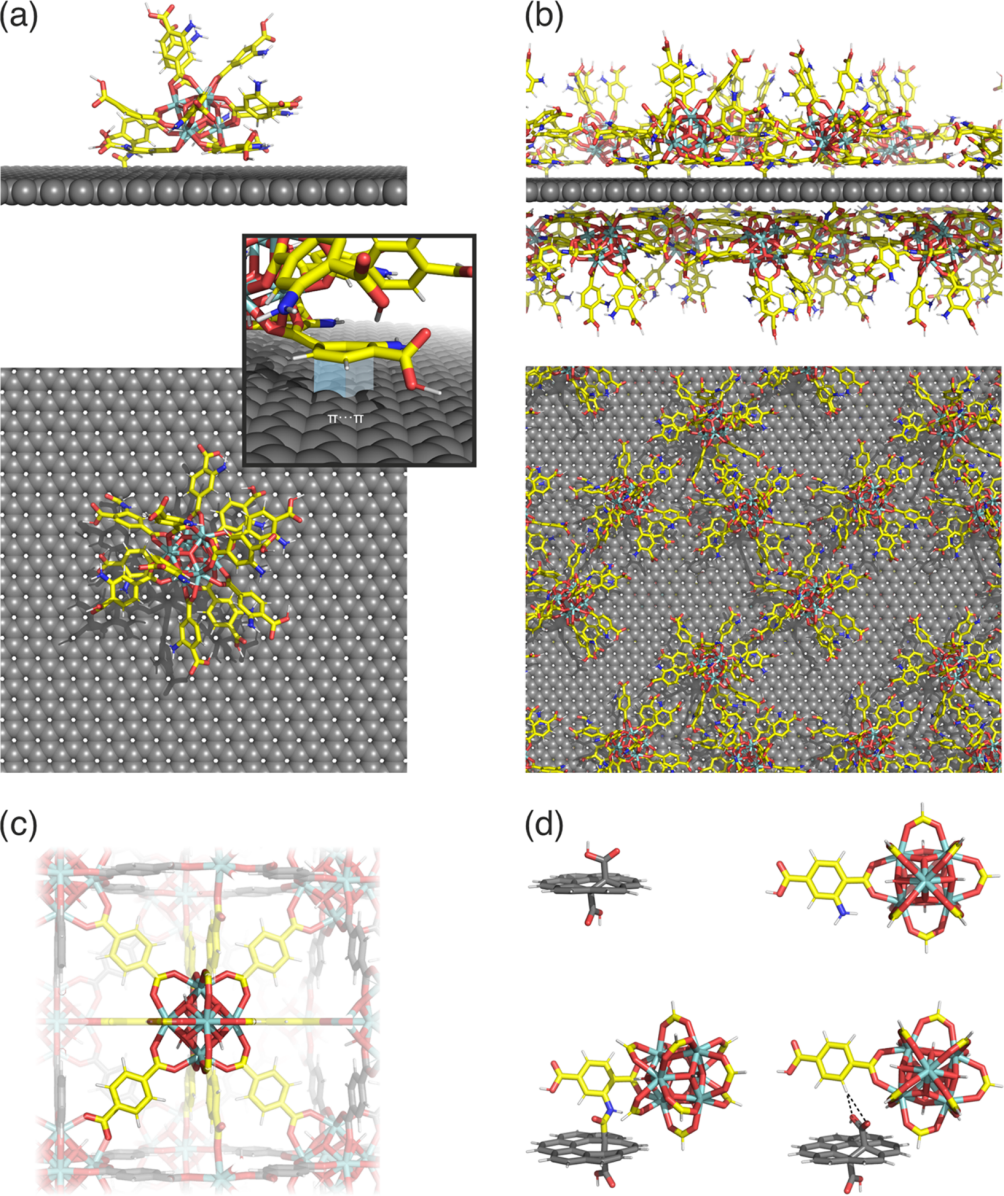
Snapshots from molecular dynamics simulation showing bonding of UiO-66-NH_2_ on graphene acid at a very sparse (a) and at greater degree of functionalization (an 8-fold concentrated system) (b). (c) Depiction of the cluster model of MOF considered in this study. (d) Optimized hybrid geometries of the Zr-based MOF with graphene acid. Carbon, hydrogen, oxygen, nitrogen, and zirconium atoms are represented as yellow, white, red, blue, and turquoise, respectively. Carbon atoms of graphene acid are represented as gray for the sake of clarity.

In our previous studies, the amide bond formation of graphene acid with amine-functionalized MOF was reported.^19,20^ In the first step of the study, the bonding of the UiO-66-NH_2_ to a GA was analyzed by means of force field based molecular dynamics (MD). Our simulation suggested a good structural compatibility of the UiO-66-NH_2_ moiety anchored on the GA surface even at higher (1.2%) coverage (Figure 1). The covalently bonded spatial MOF structure lay on the graphene surface, with its three closest terephthalate groups forming an additional π-stacking interaction with the surface. The rest of the groups retain their normal orientation with respect to the surface and remain attached to the surroundings.

The MD simulations reveal the stable hybrid structure of GA@UiO-66-NH_2_ with amide linkages. To shed more light on the mechanism of this metal-catalyzed amidation of graphene acid with the amine-functionalized MOF, DFT calculations are implemented in this study with suitable model systems. The graphene acid (GA) is modeled by coronene-(COOH)_2_ having 24 carbons, 12 hydrogens, and 2 COOH groups in the trans orientation (Figure 1d). Because of the large number of atoms in the Zr-based MOF, a cluster model containing a Zr node (consisting of Zr_6_O_4_(OH)_4_ terminated with six formate linkers) and a single connected linker (NH_2_-bdc) is selected (Figure 1c,d). Such a cluster model has been previously utilized successfully to show their interaction with graphene oxide.^18^ The geometry of the covalently linked amine-functionalized MOF with GA is optimized (Figure 1d). For comparison, the interaction of the UiO-66 MOF with GA is also considered in this study (Figure 1d). UiO-66-NH_2_ MOF contains an additional type of binding site (amino group) in comparison to UiO-66, which is responsible for covalent linking, and the carboxylic acid group of the GA can react with the amino group to form an amide bridge. The C–N bond within the amide bridge, which connects the MOF and GA in GA@UiO-66-NH_2_, is calculated as 1.372 Å. This value agrees well with the MD calculated value of 1.435 Å. Unlike the UiO-66-NH_2_ MOF, the UiO-66 MOF interacts with GA predominantly via hydrogen bonding.

**Figure 2:**
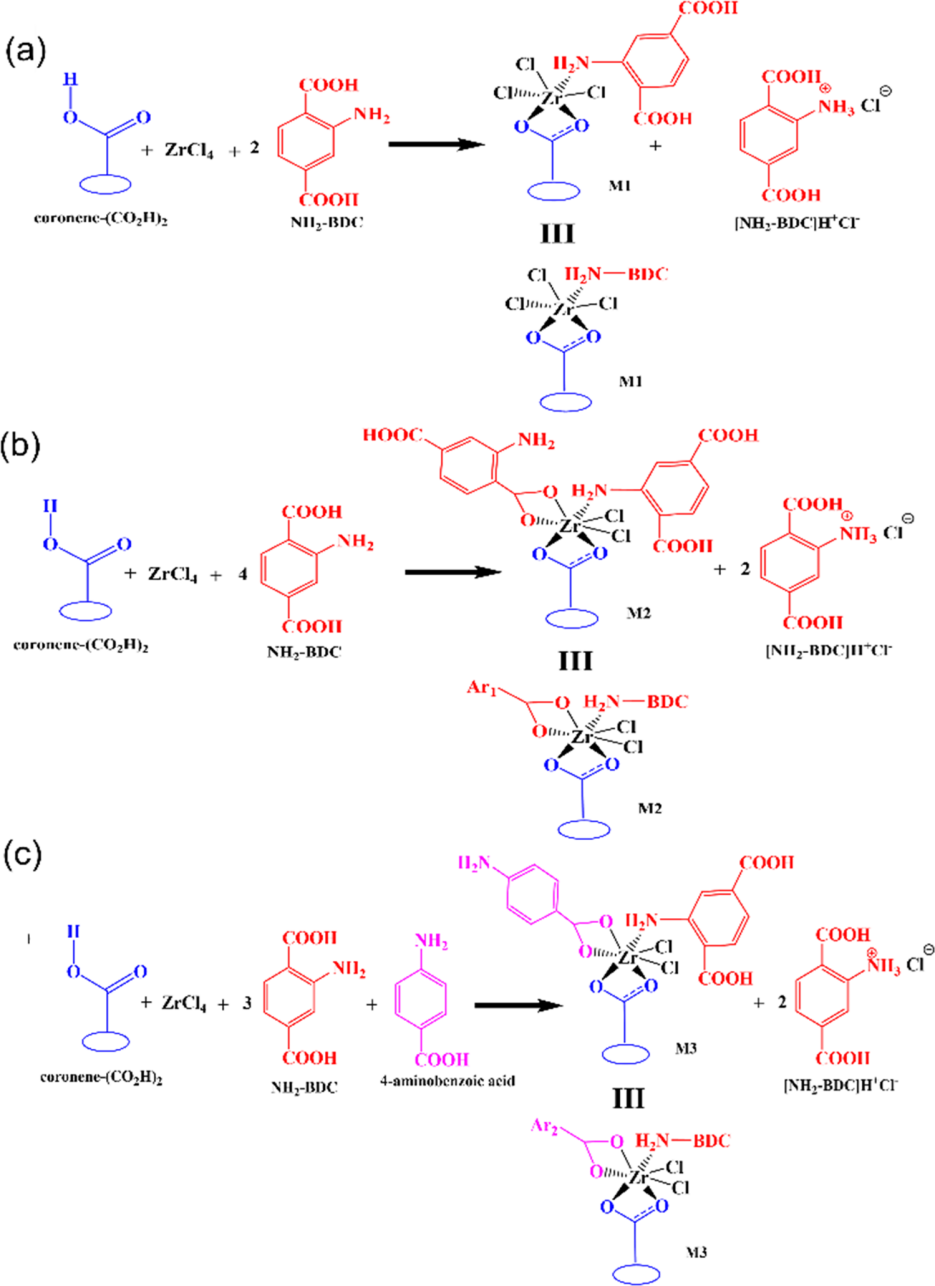
Possible starting complexes for the catalytic reactions. (a–c) Possible reaction for the generation of active catalysts M1, M2, and M3 that can serve as a starting point for the catalytic cycle.

For the zirconium-catalyzed condensation of GA and amine, we propose a mechanism in Figure 2. In this calculation, the modeled graphene acid, coronene-(COOH)_2_, ZrCl_4_, and 2-aminoterephthalic acid are considered as the initial substrates for the catalytic reaction. The coordination modulator, 4-aminobenzoic acid, which promotes the formation of missing-cluster defects, is also considered here. Zirconium generally adopts an octahedral coordination environment. Previous reports suggest that the active catalyst of Zr complex comprises up to two carboxylate ligands per Zr center.^39^

In the studied mononuclear Zr complex (M1), the carboxylate group of GA coordinates to Zr in a bidentate fashion (Figure 2a). Additionally, the amine group of 2-aminoterephthalic acid coordinates with the Zr center via an amine group. This active catalyst complex (M1) is formed by the binding of coronene-(COOH)_2_ to ZrCl_4_ and leads to the dissociation of an HCl molecule. Furthermore, the HCl molecule forms a salt complex with another 2-aminoterephalic acid (Figure 2a). The formation of the M1 complex is highly favorable, and the calculated energy of formation for the M1 complex is found to be −59.5 kcal/mol relative to the starting materials shown in Figure 2a. We extended the study by considering another Zr complex (M2) containing two carboxylates coordinated to the Zr center in a bidentate fashion (Figure 2b). It needs to be mentioned that the M2 complex contains a hepta-coordinated Zr center as zirconium centers can adopt such coordination along with commonly found octahedral coordination environment.^39,40^ The binding of two carboxylates to the Zr center produces two HCl molecules, which further form salt complexes with 2-aminoterephthalic acid. The formation of the M2 complex is also highly favorable, with a formation energy of −79.6 kcal/mol relative to the starting materials shown in Figure 2b. Similarly, 4-aminobenzoic acid also forms a hepta-coordinated Zr complex (M3) along with GA having a formation energy of −81.2 kcal/mol (Figure 2c).

**Figure 3:**
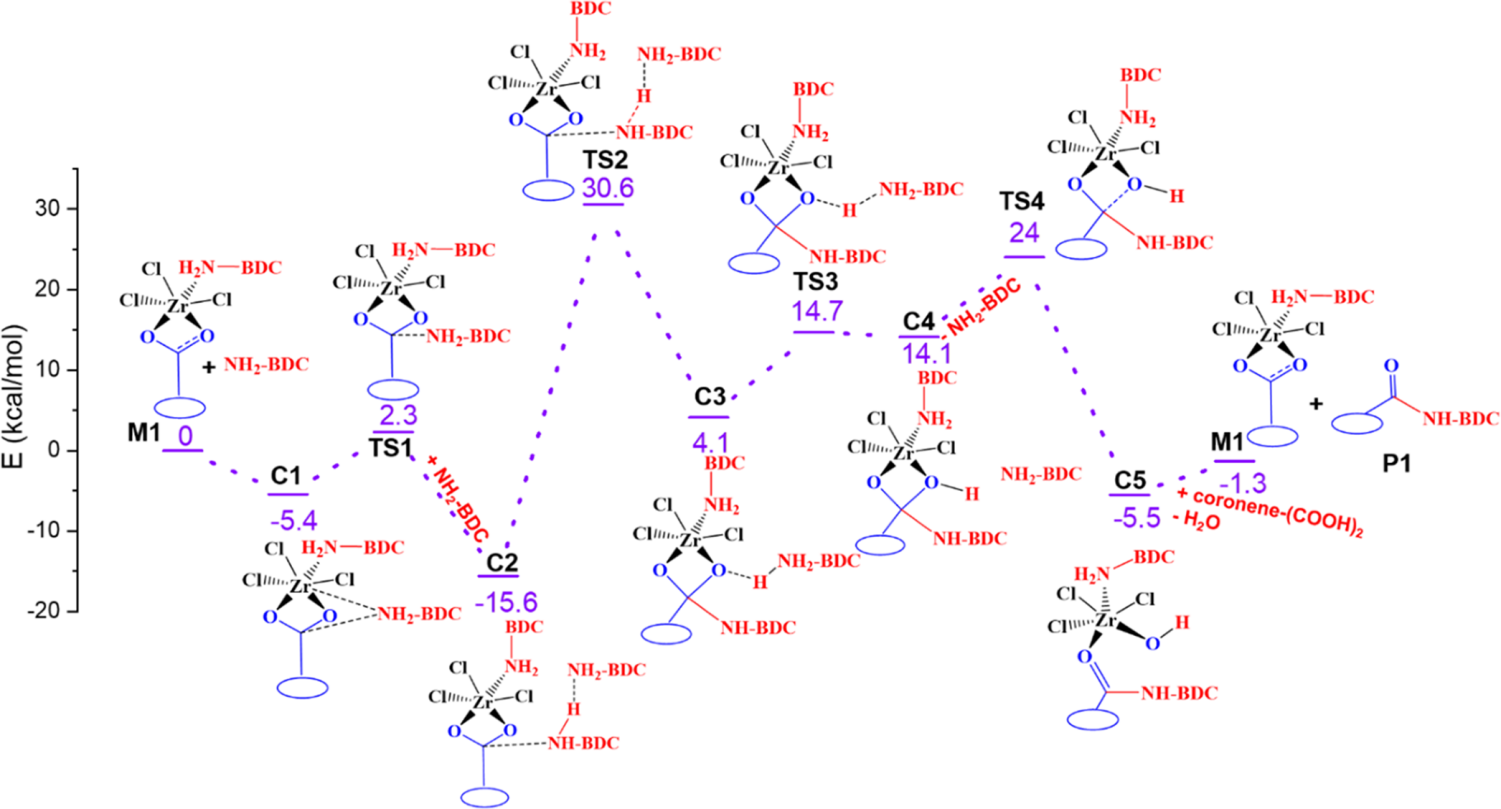
Electronic energy diagram for forming the amide product (P1) from graphene acid and UiO-66-NH_2_ proceeds by using active catalyst M1.

With these three active catalysts, we studied the amidation mechanism of the UiO-66-NH_2_ MOF with GA. Figure 3 shows the potential energy surface (PES) for the formation of amide bonds with the MOF on GA. The reaction starts with the nucleophilic attack of an external 2-aminoterephthalic acid on the carboxylate carbon center of M1 (Figures 2 and 3). During the course of the reaction, 2-aminoterephthalic acid first binds to the M1 complex noncovalently to form a van der Waals complex, C1, stabilized by 5.4 kcal/mol relative to M1. The reaction proceeds through the first transition state TS1 to the C2 complex. The amine group of an additional 2-aminoterephthalic acid stabilizes the C2 complex by forming a stable H-bond. The generated complex C2 is −15.6 kcal/mol lower in energy compared to separated reactants, M1 and NH_2_-BDC (Figure 3). Next, a proton transfer occurs to the external 2-aminoterephthalic acid via a transition state TS2 leading to C3 complex. The reaction barrier is quite high, and the calculated barrier for this step is 30.6 kcal/mol. 2-Aminoterephthalic acid is activated after deprotonation and forms the Zr-bound amide product. However, this complex (C3) is energetically less stable than the starting complexes by 4.1 kcal/mol. The transferred proton to the external 2-aminoterephthalic acid in the C3 complex forms a hydrogen bond to the oxygen of the carboxylate group (Zr–O–C). In the reaction process, the proton is transferred from external amine acid to oxygen with a transition state, TS3. This process accelerates the C–O bond cleavage step. The transition state for this step is 24.0 kcal/mol relative to the starting substrates. In this process, the C4 intermediate is energetically unstable, and the dihydroxylation occurs by attaching the hydroxyl group on the Zr Lewis acid site. The splitting of the hydroxyl group proceeds through the transition state TS4 with an energy barrier of 24.0 kcal/mol. Now the amide product (C5) is formed, which is quite stable. To release the amide product from the catalyst, a new graphene acid is added in the reaction scheme. The incoming GA protonates the hydroxyl group on the Zr Lewis acid site, which is released as a water molecule. At the end, the catalytic cycle is closed with the catalyst M1, water, and the amide product.

**Figure 4:**
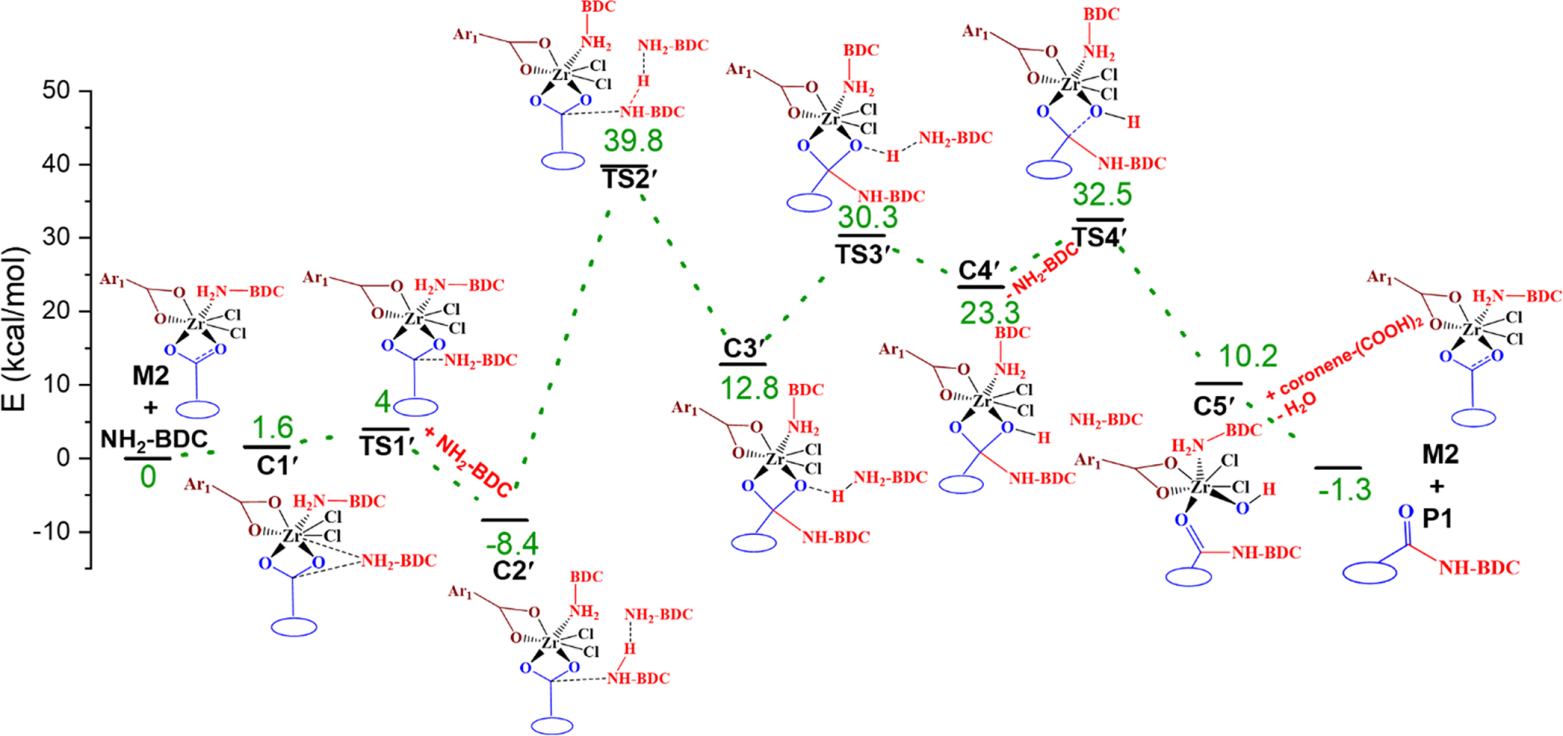
Electronic energy diagram for forming the amide product using active catalyst M2.

We have also investigated a reaction pathway wherein a hepta-coordinated Zr complex M2 is considered as an active catalyst. Figure 4 shows the reaction mechanism of the amide product starting with the formation of the active catalyst M2. Very similar pathways are obtained but with substantial differences in energy profiles (Figure 4). It is interesting to mention that the nucleophilic attack of an external amine is less favorable compared with the reaction profile with the M1 complex. The energy barrier is calculated to be higher for the M2 complex than for the M1 complex (4.0 kcal/mol vs 2.3 kcal/mol). Two 2-aminoterephthalic acid molecules stabilize the M2 complex through interaction with the Zr active site as well as intermolecular N–H...N hydrogen bonding. Furthermore, the C–O bond cleavage step also proceeds through a higher energy barrier (32.5 vs 24.0 kcal/mol), thus making the pathway less likely. The higher energy barriers are probably the result of a higher extent of steric repulsion around the Zr site as well as lowering of Lewis acidity of the Zr site. For calculations starting with the M3 complex, the energy profile has a very similar reaction step to the M2 complex (Figure S1). The calculated energy barriers for M3 are higher than those for M2 in proton transfer and C–O bond cleavage steps. Also in the reaction pathway, sterics plays an important role for higher barriers. The direct reaction of 2-aminoterephthalic acid with GA is the result of unreactive ammonium carboxylate salt formation (Figure S2). Such an ammonium salt cannot reprotonate the graphene carboxylate back to the neutral acid form and is considered to be unreactive. In light of the direct amide formation by acid catalysis, a reaction mechanism is proposed in the presence of excess acids (Figure S2).

## Conclusions

Computational studies were conducted on a hybrid structure composed of GA and the amine-functionalized UiO-66 metal–organic framework (MOF) to investigate the mechanistic pathway of the amidation process. Classical molecular dynamics simulations demonstrated the stability and geometric orientation of the covalent assemblies in the hybrid structures. Density functional theory (DFT) studies on the reaction channels highlighted the significance of additional basic amino sites present in UiO-66-NH_2_. It was observed that UiO-66 MOF, lacking the amino group, interacted with GA noncovalently. The calculated reaction profiles emphasized the crucial role of the zirconium(IV) catalyst in facilitating the amidation of nonactivated carboxylic acids in GA. These findings present novel prospects for the development of selective and straightforward covalently linked hybrid GA@UiO-66-NH_2_ materials, featuring a hierarchical porous conductive network that can be applied in gas sensing and as electrode materials for asymmetric supercapacitors.

## Supporting Information Available

Electronic energy diagrams (Figures S1 and S2) and computational methods (PDF)
