# Supplementary material for: Lewis Acid Catalyzed Amide Bond Formation in Covalent Graphene–MOF Hybrids
Source: J Phys Chem C Nanomater Interfaces. 2023 Jun 29;127(31):15454–60. doi: 10.1021/acs.jpcc.3c01821 (PMC10426341; doi:10.1021/acs.jpcc.3c01821)
Supplement: Supplementary file 1 — jp3c01821_si_001.pdf [file jp3c01821_si_001.pdf]

Lewis Acid Catalyzed Amide Bond Formation in Covalent Graphene-MOF Hybrids

Rabindranath Lo, *# Martin Pykal,# Andreas Schneemann Radek Zbořil, Roland A Fischer,* Kolleboyina Jayaramulu,* and Michal Otyepka*

Dr. Rabindranath Lo

Institute of Organic Chemistry and Biochemistry, Czech Academy of Sciences, v.v.i., Flemingovo nám. 2, 160 00 Prague 6, Czech Republic. E-mail: rabindranath.lo@uochb.cas.cz

Dr. Rabindranath Lo, Dr. Martin Pykal, Prof. R. Zbořil, Dr. Kolleboyina Jayaramulu, Prof. Michal Otyepka,

Regional Centre of Advanced Technologies and Materials, Czech Advanced Technology and Research Institute, Palacký University Olomouc, Křížkovského 511/8, Olomouc 77900, Czech Republic

E-mail: rabindranath.lo@uochb.cas.cz; jayaramulu.kolleboyina@upol.cz; michal.otyepka@upol.cz

Prof. Roland A Fischer

Chair of Inorganic and Metal–Organic Chemistry, Department of Chemistry and Catalysis Research Centre, Technical University of Munich, 85748 Garching, Germany

E-mail: roland.fischer@tum.de

Dr. Kolleboyina Jayaramulu

Department of Chemistry, Indian Institute of Technology Jammu, Jammu & Kashmir 181221, India

E-mail: jayaramulu.kolleboyina@upol.cz

Prof. Michal Otyepka

IT4Innovations, VŠB-Technical University of Ostrava, 17. listopadu 2172/15, 70800 Ostrava-Poruba, Czech Republic

E-mail: michal.otyepka@upol.cz

Dr. Andreas Schneemann

Lehrstuhl für Anorganische Chemie I, Technische Universität Dresden, Bergstr. 66, 01069 Dresden, Germany

Prof. R. Zbořil

Nanotechnology Centre, CEET, VSB – Technical University of Ostrava, 17. listopadu 2172/15, 70800 Ostrava-Poruba, Czech Republic

Methods

Molecular dynamics. All MD simulations were carried out in LAMMPS[1] (version 16 March 2018) using the Universal Force Field (UFF)[2] and a 2 fs timestep. The non-bonded potential parameters used in the simulations are listed in Table S1. As in the original parameter derivation, no partial charges were used.[2] It was shown that UFF provides a good description of several well-studied MOFs.[3] Graphene surface was modelled as periodic sheet and was kept rigid during the simulation. Initial atomic coordinates for the UIO-66 fragment was taken from Crystallography Open Database (COD).[4] MOF structures were attached to graphene surface via a carboxylic functional group (COOH). Two degrees of functionalization were considered: single functionalized graphene and system with 8 MOF moieties. Individual UIO-66-NH_2 motifs were randomly distributed over the surface on both sides. The dimensions of the box were 41.22 × 42 × 100 Å. Periodic boundary conditions were applied in all three dimensions. Pairwise interactions were computed using standard Lennard-Jones 12-6 potential with a cutoff of 12.5 Å. The system was minimized and equilibrated for 1 ns in NVT ensemble at 298 K using the Nosé-Hoover thermostat. Results were obtained from 5 ns NVT production run. Data were collected every 2 ps. Figure was prepared using PyMOL software.[5]

Table S1 Used UFF atom types and corresponding non-bonded parameters in the format of the original work of Rappé et al.[2]

Atom type	*Distance x_i (Å)*	*Energy D_i (kcal/mol)*
Zr3+4	3.124	0.069
O_R	3.500	0.060
C_R	3.851	0.105
O_3	3.500	0.060
H_	2.886	0.044
N_2	3.660	0.069

Cluster calculations. In this study, GA was modelled by coronene$(COOH)_2$ having 24 carbons, 12 hydrogens and $2CO_2H$ groups in trans orientation. All the structures considered in this study were optimized at the DFT-D3/PBE0/def2-SVP level of theory[6–8] with TURBOMOLE 7.3 suite of program.[9] The transition state optimizations were performed with Turbomole 7.3 suite of program. We followed the steps outlined in the Turbomole manual. After the optimization, the frequencies were calculated analytically using *aoforce* module. The presence of single large imaginary frequency suggests that the systems is in a transition state. Single

point energies were calculated using Grimme's advanced dispersion-corrected approach (DFT-D3) with the def2-TZVPP basis set.[10] DMF solvent was modelled by implicit solvent model (COSMO). The GA in isolated state was fully optimized whereas in the complex, only C atom and COOH group, which was involving in the reaction were relaxed while all the remaining atoms in the surface were frozen.

References:

(1) Plimpton, S. Fast Parallel Algorithms for Short-Range Molecular Dynamics. *J. Comput. Phys.* **1995**, *117*, 1–19.

(2) Rappé, A. K.; Casewit, C. J.; Colwell, K. S.; Goddard, W. A.; Skiff, W. M. UFF, a full periodic table force field for molecular mechanics and molecular dynamics simulations. *J. Am. Chem. Soc.* **1992**, *114*, 10024–10035.

(3) Boyd, P. G.; Moosavi, S. M.; Witman, M.; Smit, B. Force-Field Prediction of Materials Properties in Metal-Organic Frameworks. *J. Phys. Chem. Lett.* **2017**, *8*, 357–363.

(4) Graulis, S.; Chateigner, D.; Downs, R. T.; Yokochi, A. F. T.; Quirós, M.; Lutterotti, L.; Manakova, E.; Butkus, J.; Moeck, P.; Bail, A. Le Crystallography Open Database – an open-access collection of crystal structures. *J. Appl. Crystallogr.* **2009**, *42*, 726–729.

(5) Schrödinger, L.L.C. The PyMOL Molecular Graphics System, Version 1.8; 2015

(6) Adamo, C.; Barone, V. Toward reliable density functional methods without adjustable parameters: The PBE0 model. *J. Chem. Phys.* **1999**, *110*, 6158–6170.

(7) Weigend, F. Hartree–Fock exchange fitting basis sets for H to Rn. *J. Comput. Chem.* **2008**, *29*, 167–175.

(8) Weigend, F.; Ahlrichs, R. Balanced basis sets of split valence, triple zeta valence and quadruple zeta valence quality for H to Rn: Design and assessment of accuracy. *Phys. Chem. Chem. Phys.* **2005**, *7*, 3297–3305.

(9) TURBOMOLE V7.3 2018, a development of University of Karlsruhe and Forschungszentrum Karlsruhe GmbH, 1989–2007, TURBOMOLE GmbH, since 2007; available from http://www.turbomole.com.

(10) Grimme, S.; Antony, J.; Ehrlich, S.; Krieg, H. A consistent and accurate ab initio parametrization of density functional dispersion correction (DFT-D) for the 94 elements H-Pu. *J. Chem. Phys.* **2010**, *132*, 154104.

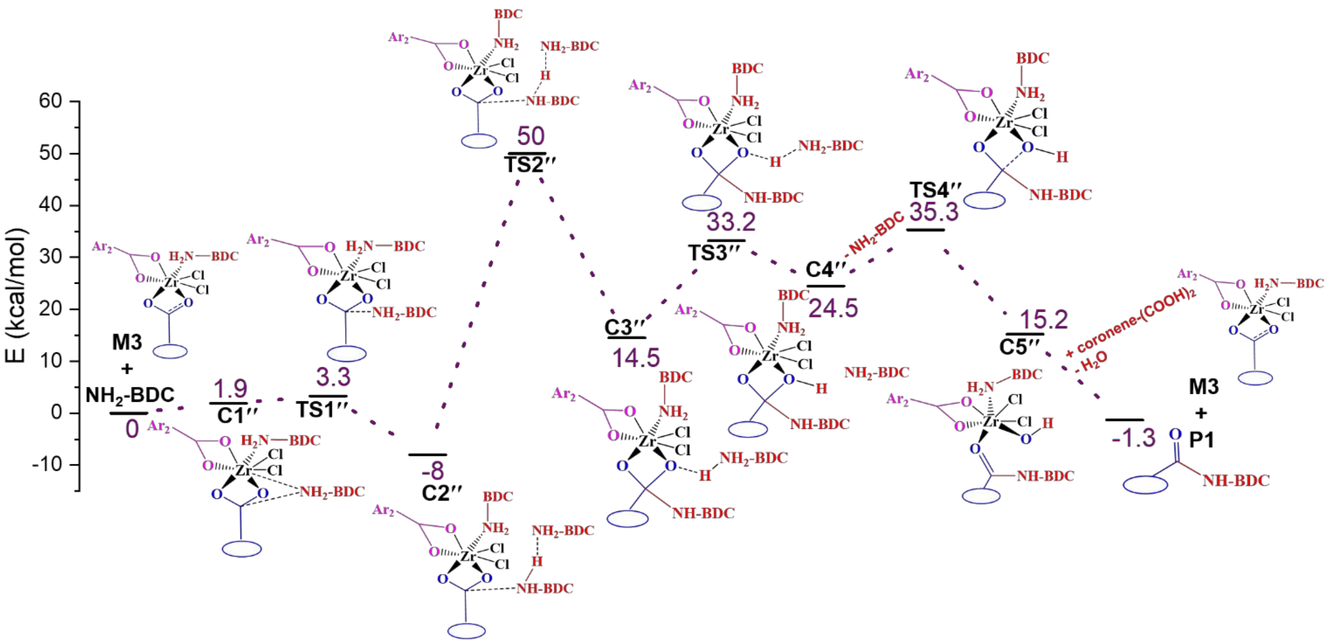

Figure S1 Electronic energy diagram for forming the amide product using active catalyst M3.

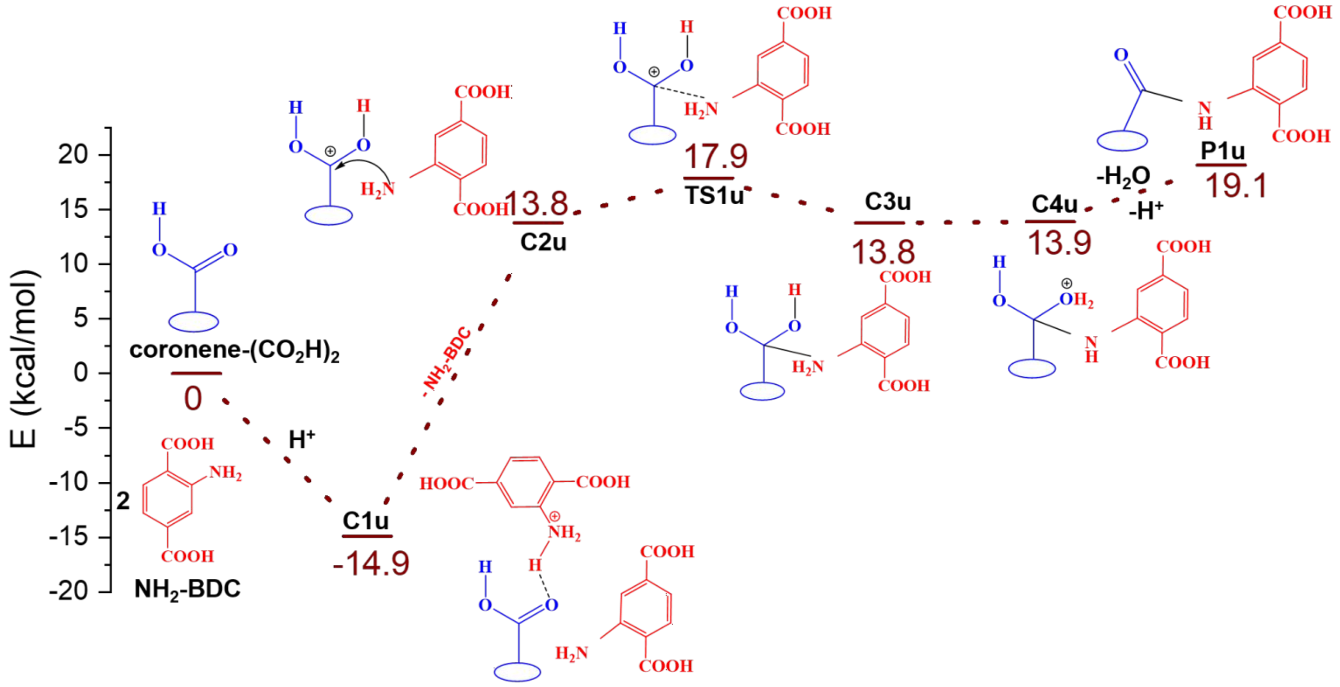

Figure S2 Electronic energy diagram for forming the amide product using acid catalyst.
